# P22 How do we improve gentamicin prescribing and reviewing within colorectal wards—a quality improvement project

**DOI:** 10.1093/jacamr/dlae136.026

**Published:** 2024-08-23

**Authors:** Lauren Smith, Espe Palenzuela, Rebekah Sutherland

**Affiliations:** Western General Hospital, Edinburgh, UK; Western General Hospital, Edinburgh, UK; Western General Hospital, Edinburgh, UK

## Abstract

**Objectives:**

A retrospective study investigating gentamicin prescribing and reviewing, focusing on Trak short code ‘\gent’, across colorectal wards at Western General Hospital, Edinburgh.

**Methods:**

A focussed questionnaire regarding NHS Lothian gentamicin prescribing charts and Trak short code ‘\gent’ was placed around targeted wards in Western General Hospital in November 2023. A retrospective search of HEPMA data including all patients who had received gentamicin in Colorectal wards between 18 August 2023 and 18 December 2023. A total of 100 patients, 25 from each ward, were randomly selected. Trak data was analysed using the search terms; ‘gentamicin’, ‘IVOS’ and ‘\gent’. Patient data including the number of gentamicin doses received, indications for gentamicin prescribing, evidence of documentation of gentamicin reviewing and on what day of the course, and the use of the ‘\gent’ Trak short code was gathered.

**Results:**

Questionnaire results: 33 responses, 75.8% (25) felt the NHS Lothian gentamicin charts are not well designed, 72.7% (23) did not know ‘\gent’ existed. From the HEPMA search, 100 patients were included, of which 59% were male. The median age was 67 years (19–90). The average gentamicin dose received was 3.8 doses, while 52% of patients received 5 doses. Indication of gentamicin; 58% intra-abdominal infection. Sixty-five percent had documented discussion of gentamicin reviewing, 69.2% of these only occurred on the day of dose 5. Thirty-five percent had no documentation of gentamicin reviewing; 80% of the course length prompts occurred on Day 5. There was no documented discussion on Day 3 of gentamicin, as prompted by the NHS Lothian gentamicin chart. Overall use of \gent was 2%.

**Conclusions:**

(i) The Trak short code ‘\gent’, designed to help make prescribing easier, is not being used. (ii) A large proportion of clinical staff are unaware of ‘\gent’. (iii) Clinical teams do not routinely document their gentamicin reviewing. (iv) Delayed gentamicin doses continue to be given due to lack of communication and lack of documentation. Since completing the search, an informative poster has been placed around the same wards to highlight ‘\gent’ and its uses. Further data will be collated, and a second cycle of the project will involve another retrospective HEPMA search and study of gentamicin patients and the use of ‘\gent’ with the aim of an increase in use and discussion of the review process.

